# Severe pancytopenia at the presentation of Imerslund-Gräsbeck syndrome in a 23-month-old Italian boy

**DOI:** 10.1186/s13052-024-01759-x

**Published:** 2024-09-18

**Authors:** Francesca Di Sario, Francesca Piloni, Francesco Gasparini, Eleonora Serpetti, Barbara Bruschi, Paola Coccia, Maria Elena Lionetti, Simona Gatti

**Affiliations:** 1 Department of Pediatrics, Polytechnic University of Marche, via Corridoni, Ancona, 60123 Italy; 2 Department of Pediatric Haematology and Oncology, Azienda Ospedaliera delle Marche, via Corridoni 11, Ancona, 60123 Italy

**Keywords:** Imerslund-Gräsbeck syndrome, Pancytopenia, Vitamin B12, Amnionless gene, Cobalamin deficiency, Proteinuria

## Abstract

**Background:**

Imerslund-Gräsbeck syndrome (IGS) is a rare autosomal recessive disorder characterized by megaloblastic anemia due to selective cobalamin malabsorption and benign proteinuria. IGS is caused by a disfunction of the cubam receptor, which mediates the reabsorption of cobalamin in the ileum and the reuptake of albumin in renal proximal tubules.

**Case presentation:**

We describe the case of a 23-month-old-italian infant presenting with severe pancytopenia and failure to thrive in whom the diagnosis of IGS was made and vitamin B12 replacement therapy was resolutive. Genetic analysis (NGS with CNV analysis including 214 genes involved in bone marrow failure and anemia), showed the presence of two pathogenetic variants in the *AMN* gene (c-208–2 A > G and c.1006 + 34_1007-31del). These variants have been previously described in the literature, but their combination has never been reported.

**Conclusions:**

Imerslund-Gräsbeck syndrome should be considered in the differential diagnosis of children with severe pancytopenia even in those without neurological involvement. This case emphasizes the importance of an early diagnosis and prompt treatment, to prevent irreversible neurological injury.

**Supplementary Information:**

The online version contains supplementary material available at 10.1186/s13052-024-01759-x.

## Background

Imerslund-Gräsbeck syndrome (IGS) is a rare autosomal recessive disorder characterized by megaloblastic anemia, persistent but benign proteinuria (present in about 50% of patients), failure to thrive, and other nonspecific symptoms, such as recurrent gastrointestinal or respiratory infections, pallor, and weakness. Neurological involvement can be observed but it is usually mild [1].

IGS is caused by mutations in cubilin (*CUBN*) or amnionless (*AMN*) gene, which encode for the subunits of the heteromeric receptor known as cubam, highly expressed both in epithelial brush borders of the distal small intestine (which mediates the uptake of cobalamin) and renal proximal tubules (for the reuptake of albumin) [2].

The estimated prevalence of IGS is less than 6 cases per million inhabitants, and about 300 cases have been reported worldwide [3]. No information about the epidemiology of IGS in Italy is available, where only sporadic cases are described [4, 5]. The disease often manifests before the 5th year of life, though it has been diagnosed in patients from other age groups. Lifelong vitamin B12 supplementation is the only available treatment for IGS.

We herein describe the case of a 23-month-old-italian boy presenting with pancytopenia, severe cobalamin deficiency, and albuminuria, for whom the diagnosis of IGS was clinically suspected and subsequently genetically confirmed. Specifically, a NGS analysis including all known genes involved in bone marrow failure and anemia was performed, and a heterozygous compound pathogenetic variant in the *AMN* gene was detected.

### Case presentation

A 23-month-old boy was referred to the Pediatric Emergency Department with a 2–3-week history of evening fever (37.6–37.7 °C), loss of appetite, fatigue, recurrent respiratory infections, and failure to thrive.

**Fig. 1:**
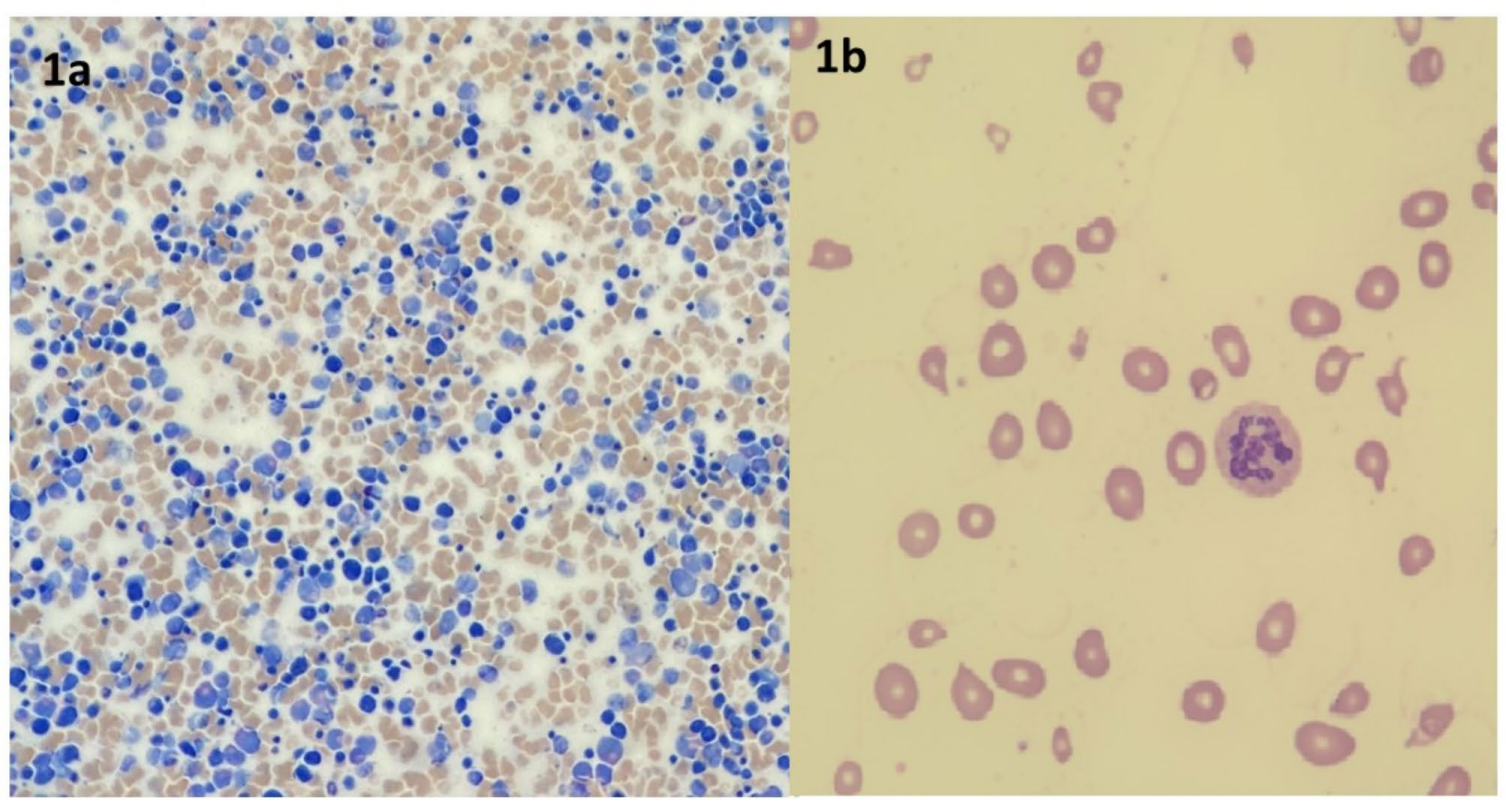
**A** Bone marrow smear: polymorphic bone marrow with a high prevalence of erythroid precursors. **B** Peripheral blood smear: megaloblastosis, a single hypersegmented neutrophil, poikilocytosis, and anisocytosis

Physical examination revealed pale skin and palpable liver and spleen. There were no signs of airway or gastrointestinal infections, and neurological status was normal. Family history was uneventful. The child’s medical history revealed that he was born at term, after uncomplicated pregnancy and delivery. He was breastfed until one year of life and his mother had been following a various and complete diet. His growth showed a linear trend from birth but in the last six months, his weight and length gain started to slow down from 80th to 50th percentile and from 97th to 75th, respectively (WHO child growth standards were used) [6]. Initial blood investigations showed pancytopenia with severe macrocytic anemia (hemoglobin 4.5 g/dl, mean corpuscular volume 102 fl., mean corpuscular haemoglobin 34.1 pg, mean corpuscular haemoglobin concentrations 33.3 g/dl, reticulocytes count 52.100/mmc, white blood cells and platelets count were 3.99 × 103/mm3 and 70 × 103/mm3 respectively). Because of these findings, he was initially admitted to the Pediatric Hematology Department and evaluated for the suspicion of acute leukemia. A transfusion of packed red blood cells was required. Bone marrow aspirate did not show evidence of blasts and other white cell line abnormalities, excluding thus blood cancer. Conversely, abnormal maturation and proliferation of red cell precursors was evident (Fig. 1a). Therefore, further laboratory investigations were performed, showing increased levels of unconjugated bilirubin (1.60 mg/dl), increased lactate dehydrogenase (LDH > 4000 U/l), as well as decreased ones of haptoglobin (< 8 mg/dL) and severe vitamin B12 deficiency (0.09 ng/Ml; n.v. 0,25 − 1,10). The assessment of peripheral blood smear revealed megaloblastosis, with the evidence of hypersegmented neutrophils, poikilocytosis and anisocytosis (Fig. 1b). Based on these findings, a diagnosis of megaloblastic anemia (with leucopenia and thrombocytopenia) due to vitamin B12 deficiency was made. Therefore, intramuscular replacement therapy with cyanocobalamin (1000 µg) was started, leading to normalizing vitamin B12 serum levels and resolution of clinical symptoms after a few days. The number of leukocyte and platelets gradually increased with complete normalization of both counts after 15 days. A careful dietary interview revealed a variegate and adequate dietary intake. This finding and the normality of other nutritional blood parameters (albumin, folate, ferritin, vitamin D, A and E) excluded the possibility of a nutritional vitamin B12 deficiency. Gastroenterological work-up (including negative celiac markers, anti-gastric parietal cells and anti-intrinsic factor antibodies, fecal elastase, fecal calprotectin, and abdominal ultrasound) ruled out other common causes of vitamin B12 malabsorption.

Meanwhile, mild-moderate proteinuria (albuminuria 70 mg/dL) with normal kidney function was detected. To better characterize the origin of vitamin B12 deficiency a metabolic screening was performed, showing increased levels of homocysteine (Hcyst) and methylmalonic acid (MMA) in serum and urine, as expected in inborn errors affecting cobalamin uptake or metabolism.

The simultaneous presence of severe vitamin B12 deficiency, proteinuria, and high levels of Hcyst and MMA was suggestive for the diagnosis of Imerslund-Gräsbeck syndrome.

Genetic analysis (NGS-based CNV analyses including 214 genes involved in bone marrow failure and anemia) revealed the presence of a heterozygous compound pathogenetic variant (c-208–2 A > g in the splice acceptor site of exon 4 and c.1006 + 34_1007-31del) in the *AMN* gene, consistent with the diagnosis of autosomal recessive Imerslund-Grasbeck syndrome type 2. *CUBN* gene was also analyzed but no mutations were detected. The family genetic testing was proposed but both parents refused genetic analysis. A genetic counseling was provided, although a prognostic evaluation of the recurrence risk for the couple was not possible.

Treatment was continued with an intramuscular monthly injection of vitamin B12 (500 µg) and periodic clinical and biochemical follow-up (Table 1).

**Table 1:** The main laboratory findings at admission and during the follow-up

	At admission	During hospitalization (pre-treatment)	At discharge	After 6 months	At last Follow-up (36 months)
*White blood cells/mmc*, *(n.v. 6–17)*	3.990	3.960	7.570	7.110	6.470
*Haemoglobin*,* g/dl* *(n.v. 11*,*5–13)*	4,5	8,4	10	13,7	13,2
*Mean corpuscular volume*,* fl. (n.v. 75–87)*	102	93	102	86	87
*Platelets cells/mmc*, (n.v. 250–550 *10 ^*3*^ *)*	73.000	43.000	106.000	213.000	88.000
*Vitamin B12*,* ng/ml* *(n.v. 0*,*25 − 1*,*10)*	0,09	n.a.	4,04	2,44	0,89
*Plasmatic Metil Malonic Acid*,* mmol/l (n.v. <2)*	24	n.a.	n.a.	n.a.	1,1
*Plasmatic homocysteine*, *mcmol/l (n.v. 5–15)*	54,67	40,8	n.a.	n.a.	8,5
*Proteinuria*,* mg/dl* *(n.v.1–14)*	30	70	20	n.a.	15

n.a.= not available

At the last follow-up (36 months) the child has regular neurological development and somatic growth (weight and height on the 75th percentile). Blood count was normal except for moderate thrombocytopenia. To further define this aspect, appropriate laboratory investigations were carried out; anti-platelets antibodies were negative excluding immune thrombocytopenia, whereas child’s medical history revealed a recent COVID-19 infection, suggesting a COVID-19 related thrombocytopenia. The platelet count, indeed, normalized after one week.

## Discussion and conclusions

We have herein described the case of a 23-month-old boy who presented at onset pancytopenia associated with benign proteinuria and failure to thrive. The diagnosis of IGS was initially suspected on a clinical basis and subsequently genetically confirmed. The key findings to suspect the diagnosis of IGS in our patient were mild low molecular weight proteinuria, with normal renal function, associated with megaloblastic anemia due to severe vitamin B12 deficiency. In addition, according to the published cases, the age of onset was typical [7].

Vitamin B12 deficiency can present with severe symptoms in children, particularly failure to thrive, developmental delay or regression, hypotonia, lethargy, tremors, and feeding issues. Neurological symptoms are related to the fundamental role of Vitamin B12 in the development and myelination of the central nervous system. Hematological abnormalities include macrocytic anemia, leuko, and thrombocytopenia, and all of them are reversible manifestations [8].

Detecting the etiology of Vitamin B12 deficiency in children is a diagnostic challenge, due to the wide variety of possible presentations and causes. Reduced intake is the most frequent cause of deficiency in newborns and children; typically, the defect is related to prolonged/exclusive breastfeeding in an infant of a mother with vitamin B12 deficiency but also secondary to the adoption of selective diets among children of all ages that is becoming more and more frequent in Western countries. Conversely, in the past, the incidence of vitamin B12 deficiency was higher in developing countries with few economic and social resources but rare in the Western world [9].

Moreover, it is important to emphasize the higher risk of neural tube defects related to maternal vitamin B12 deficiency and folate status. In this context, the role of perinatal physicians is crucial in preventing birth defects of the central nervous system [10].

Other causes of early-onset deficiency include pernicious anemia, long therapy with proton pump inhibitors, cystic fibrosis due to pancreatic insufficiency, and malabsorption. Vitamin B12 malabsorption can be the result of different mechanisms: surgical resection, Inflammatory Bowel Disease involving the ileum (such as Crohn’s Disease), small intestinal bacterial overgrowth, and genetic diseases including inherited deficits of intrinsic factor as well as Imerslund-Gräsbeck disease [11].

IGS is genetically determined; mutations of two different genes are involved in the pathogenesis of the disorder: amnionless (AMN) and/or cubilin (CUBN) gene, located at chromosome 14 and 10, respectively. These genes encode for the two components of the Cubam receptor for the intrinsic factor (IF)-cobalamin (Cbl) complex present in the ileum, whose function is to permit the absorption of vitamin B12, and of the receptor that mediates the reuptake of albumin in proximal tubule of the kidney. Cases of IGS have been sporadically described in the literature. A recent review has identified a total of 456 cases described in previous reports [6]. Interestingly most of the cases are reported in Turkey (22%), Finland (15%), Norway (7%), Tunisia (6%) and USA (6%). The condition has been occasionally described in subjects of Caucasian origin, and only two previous cases have been reported in children of Italian origin [4, 5].

To confirm the diagnosis of IGS in our patient, after an extensive diagnostic work up which ruled out the most common causes of vitamin B12 deficiency, a NGS analysis was quickly performed. This sequencing technology was chosen since it provides high diagnostic rate and allows a rapid genetic definition of many monogenic conditions [12–14]. All genes involved in bone marrow failure and anemia including the ones which mediate vitamin B12 absorption (e.g. *CUBN* and *AMN* genes) were studied performing NGS analysis; sequencing of the *AMN* gene revealed the presence of two heterozygous pathogenetic variants (c-208–2 A > g and c.1006 + 34_1007-31del) consistent with the diagnosis of autosomal recessive Imerslund-Grasbeck syndrome type 2. Both these variants have previously been described in the literature, but such compound heterozygous mutation has never been reported. The c.208–2 A > G mutation causes the complete skipping of exon 4 (88 bp) with a subsequent frameshift and a premature termination codon in exon 6. The variant c.1006 + 34_1007-31del is a 15 bp deletion in intron 9 of the *AMN* gene, and results in the skipping of exon 9 introducing a premature termination codon [15]. Each mutation retrieved in our case is reported in combination with a different one in the previous two Italian case reports [4, 5]. The description of further cases may be useful to better characterize the genetic and clinical profiles of the disease and to clarify genotype-phenotype correlations.

According to the literature and in line with the clinical course of our patient, the prognosis of IGS is good if the disease is promptly recognized and replacement therapy is started as soon as possible [16].

In conclusion, Imerslund-Gräsbeck syndrome should be considered in children with severe pancytopenia even in those without neurological involvement. Our report emphasizes the importance of an early diagnosis to prevent irreversible neurological injury.

## Electronic supplementary material

Below is the link to the electronic supplementary material.

Supplementary Material 1

## Data Availability

The datasets used and/or analyzed during the current study are available from the corresponding author on reasonable request.

## Abbreviations

IGS: Imerslund Grasbeck syndrome
CUBN: Cubilin
AMN: Amnionless
HCYST: Homocysteine
MMA: Methylmalonic acid
