# Supplementary material for: Severe pancytopenia at the presentation of Imerslund-Gräsbeck syndrome in a 23-month-old Italian boy
Source: Ital J Pediatr. 2024 Sep 18;50:186. doi: 10.1186/s13052-024-01759-x (PMC11411748; doi:10.1186/s13052-024-01759-x)
Supplement: Supplementary file 1 — Supplementary Material 1 [file 13052_2024_1759_MOESM1_ESM.docx]

Some further minor changes are now needed before publication.

*We kindly thank both the editor and the reviewers for further revision of our manuscript and final acceptance. We have addressed all the comments and modified the paper according to the suggestions (change are highlighted in green).*

Abstract

"have been previously described in the literature"

key words: amnionless gene or AMN (in italics) gene

*Thanks for the suggestions, we have changed the abstract and keywords accordingly*

Main manuscript

Background

age groups, use space

"All known genes involved in bone marrow failure..." (add known)

"a heterozygous compound pathogenic variant in the AMN gene was detected"

Case presentation

from 80th to 50th percentile and from 97th to 75th, respectively (add the reference relating to WHO growth standards)

delete "levels" after haptglobin (as it is a repetition)

transcribe Grasbeck correctly

"Genetic analysis...revealed the presence of a heterozygous compound pathogenic variant (c.208-2A>G...) in the AMN gene, consistent with the diagnosis (remove genetic)..."

Remove "significant" before mutations (relating to CUBN gene)

"Although a prognostic evaluation of the recurrence risk for the couple was not possible"

75th percentile

"The platelets count, indeed, normalized after one week"

Discussion

"including the ones which mediate (and not who)..."

remove "an" before NGS analysis

a heterozygous compound pathogenic variant A>G, consistent with the diagnosis...(remove genetic)"

remove the sentence "All together these 3 cases...in the Italian population", as we don't know if these 3 cases may subtend or not a higher prevalence of these variants in our population

*Thanks for the comments, all the suggestions have been incorporated*

Figure 2a/b , this figure is not clear, as it does not easily give the idea to the reader about the different growth of the patient between diagnosis and start of therapy, please highlight or remove

Figure 2b caption, remove the full stop after "infant"

*We agree that picture 2 was not very informative, we have removed from the manuscript*

please among the very few references cited, please add at least the following ones referring to the clinical relevance of molecular genetics diagnostic investigations, as well as to the complex pathogenic interaction between genetic (specifically if sustained by heterozygous compound variants like those currently detected) and epigentic factors in the determinism of congenital diseases, similarly to that here described:

Cardio-facio-cutaneous syndrome and gastrointestinal defects: report on a newborn with 19p13.3 deletion including the MAP2K2 gene. Gregorio Serra, Sofia Felice, Vincenzo Antona, Maria Rita Di Pace, Mario Giuffrè, Ettore Piro, Giovanni Corsello. Ital J Pediatr 2022 May;48:65.

Infant developmental profile of Crisponi syndrome due to compound heterozygosity for CRLF1 deletion. Schierz IAM, Serra G, Antona V, Persico I, Corsello G, Piro E. Clin Dysmorphol. 2020;29(3):141-143.

2q13 microdeletion syndrome: report on a newborn with additional features expanding the phenotype. Ettore Piro,, Gregorio Serra,, Mario Giuffrè, Ingrid Anne Mandy Schierz, Giovanni Corsello. Clin Case Rep. 2021;9:e04289.

*Thanks again for the suggestion, we have incorporated these citations as new references*
